# Clinical presentation and in-hospital outcomes of intraoperative red blood cell transfusion in non-anemic patients undergoing elective valve replacement

**DOI:** 10.3389/fcvm.2022.1053209

**Published:** 2022-11-22

**Authors:** Ren Zhou, Dewei Qian, Haiqing Li, Zhe Wang, Sheng Shi, Feng Shen, Lin Cheng, Dicheng Yang, Min Yu

**Affiliations:** ^1^Shanghai Institute of Hematology, State Key Laboratory of Medical Genomics, National Research Center for Translational Medicine at Shanghai, Ruijin Hospital, Shanghai Jiao Tong University School of Medicine, Shanghai, China; ^2^Department of Cardiovascular Surgery, Shanghai General Hospital, Shanghai Jiao Tong University School of Medicine, Shanghai, China; ^3^Department of Cardiovascular Surgery, Ruijin Hospital, Shanghai Jiao Tong University School of Medicine, Shanghai, China

**Keywords:** valve replacement, non-anemia, transfusion, iron deficiency, hypoxemia

## Abstract

**Background:**

Intraoperative transfusion is associated with adverse clinical outcomes in cardiac surgery. However, few studies have shown the impact of intraoperative red blood cell (RBC) transfusion on non-anemic patients undergoing cardiac surgery. We assessed the in-hospital clinical outcomes of non-anemic patients undergoing isolated valve replacements and investigated the predictors associated with intraoperative RBC transfusion.

**Methods:**

We enrolled 345 non-anemic patients undergoing isolated valve replacements in our department from January 2015 to December 2019. The patients were stratified by the receipt of intraoperative RBC transfusion. Baseline characteristics were compared between groups and multiple logistic regression was used to identify the predictors for intraoperative RBC transfusion. The association between intraoperative RBC transfusion and in-hospital outcomes was also evaluated.

**Results:**

Intraoperative RBC transfusion developed in 84 of the 345 enrolled patients (24.3%). Three independent predictors for intraoperative RBC transfusion of non-anemic patients undergoing isolated valve replacements were identified by multivariate logistic analysis, including female, iron deficiency and hemoglobin level. When the two groups were compared, a significant tendency of higher in-hospital mortality (6.0% vs. 1.1%, *P* = 0.033) and higher incidence of postoperative hypoxemia (9.5% vs. 2.7%, *P* = 0.007) were observed in the intraoperative RBC transfusion group. After adjustment, the presence of intraoperative RBC transfusion was associated with an increase in postoperative hypoxemia (OR = 3.36, 95% CI: 1.16–9.71, *P* = 0.026).

**Conclusion:**

Intraoperative RBC transfusion was associated with poorer clinical outcomes in non-anemic adults undergoing isolated valve replacements, which significantly increased the risk of postoperative hypoxemia. The independent predictors of intraoperative RBC transfusion, such as iron deficiency and female, were identified, which may be helpful for risk assessment and perioperative management.

## Introduction

The patients proceeding to cardiac surgery have a high requirement for allogeneic transfusion perioperatively (1, 2). Particularly, intraoperative transfusion is beneficial to maintain hemostasis, correct dysfunctional coagulation, and improve tissue perfusion and oxygenation in the case of massive blood loss during cardiac surgery, but it is also associated with increased postoperative complications, such as infection, acute lung injury, and cerebrovascular adverse events (3–5). Importantly, intraoperative transfusion of red blood cell (RBC) in patients undergoing cardiac surgery exceeds 40% according to previous retrospective studies, influencing significantly on complications and prognosis (6, 7). Recently, the anemic cardiac surgery patients have been reported to be more likely to get transfusion perioperatively (8–11). Indeed, anemia is common among the patients undergoing cardiac surgery, which accounts for more than 20% of the operative patients (12). However, there are few studies focusing on association between intraoperative RBC transfusion and prognosis in non-anemic patients, treated as an ignored entity, undergoing cardiac surgery.

We aimed to assess clinical characteristics of intraoperative RBC transfusion and explore the impact of intraoperative RBC transfusion on the in-hospital outcomes in non-anemic patients undergoing valve replacement surgery, providing further evidence on the clinical strategies of perioperative blood management for the non-anemic patients.

## Materials and methods

### Ethics statement

The study protocol was reviewed and approved by the ethical committees of Shanghai General Hospital (Approval number: 2020KY223). All the research procedures were carried out in accordance with relevant guidelines and regulations. The need for informed consent from the patients was waived by the ethical committees of Shanghai General Hospital because the data were recruited from electronic medical records and reported without personal identifiers.

### Study design and population

From January 2015 to December 2019, the consecutive adult patients aged from 18 to 80 years old who underwent elective valvular replacements, including mitral valve replacement, aortic valve replacement, and tricuspid valve replacement, in Shanghai General Hospital were enrolled in the study. The exclusion criteria of this study consisted of the following: (1) hemoglobin ≤13 g/dl for males and hemoglobin ≤12 g/dl for females, (2) the presence of coronary surgery, aortic surgery or ablation surgery, (3) re-operation of valvular surgery, (4) left ventricular ejection fraction (LVEF) < 35%, and (5) massive transfusion.

### Clinical practice

Median sternotomy served as a surgical approach in all surgeries. Traditional aortic cannulation was established through the ascending aorta, while femoral artery cannulation was chosen if needed. A balloon venous drainage tube was inserted through the right atrium when isolated aortic valve replacement was performed, while vena cava cannulation was established in other valve surgeries. Cold blood cardioplegia (4°C) anterograde perfusion and coverage with ice were used for myocardial arrest protection, with a moderate systemic temperature (28–30°C). Mitral valve surgery was performed through the atrial septal approach, and aortic surgery was performed through the transverse aortic incision. The type of valvular prosthesis was decided according to the patients’ age and comorbidities.

During the surgery, the cell savers (3000P, Jingjing, Beijing, China) routinely collected, processed, and returned salvaged blood to the patient during surgery. Principally, the criterion of intraoperation transfusion of RBC in our institution was hemoglobin <8.0 g/dl or hematocrit <25% after cardiopulmonary bypass (CPB), which was consistent with the guidelines issued by American Association of Blood Banks (13). Otherwise, if mixed venous blood oxygen saturation dropped to 60% or below even if hemoglobin reached the above standards, a certain number of RBCs could be administered after excluding other factors such as increased oxygen consumption, low cardiac output or hypoperfusion accordingly (14). Fresh frozen plasma (FFP) transfusion was in accordance with blood loss. The transfusion of cryoprecipitate and platelet varied from the surgeons according to intraoperative coagulation and bleeding.

### Data collection

Baseline characteristics such as demographic information, past medical history, laboratory test, electrocardiogram, and echocardiography results were obtained from their medical records of our institution, including the patients’ age, gender, body mass index (BMI), smoking history, drinking history, diabetes mellitus, hypertension, cerebrovascular disease, atrial fibrillation, New York Heart Association (NYHA) class, left ventricular ejection fraction (LVEF), prothrombin time (PT), activated partial thromboplastin time (APTT), thrombin time (TT), alanine transaminase (ALT), estimated glomerular filtration rate (eGFR), hemoglobin A1c (HbA1c), fibrinogen, platelet count, hemoglobin, albumin, hematocrit, total bilirubin, serum ferritin and transferrin saturation. Surgical types, cardiopulmonary bypass time (CPBT), aortic cross-clamp time (ACCT) and intraoperative blood product transfusion were recorded from anesthesia note. Postoperative data was as follows: requirement for postoperative RBC or FFP transfusion, in-hospital mortality, duration of mechanical ventilation, acute kidney injury (AKI), adverse cardiovascular and cerebrovascular events (MACCE), postoperative hypoxemia, nosocomial infection, and re-exploration for bleeding.

### Definitions

Of note, it has been shown that massive hemorrhage is likely to be associated with huge trauma, abnormal anatomy, or operation techniques, which will lead to poor prognosis of cardiac surgery (15, 16). Therefore, these kinds of patients were excluded from our research to offset bias. Actually, there are several different definitions of massive transfusion, such as a transfusion of more than 1 entire blood volume in 24 h, or administration of ≥10 units (U) of RBCs to a patient within 24 h (17, 18). In our study it was defined as administration ≥5U RBCs within 4 h (19, 20). Iron deficiency was defined by a serum ferritin level <100 ng/ml or <300 ng/ml in association with transferrin saturation <20% (21). Postoperative hypoxemia was defined as oxygenation impairment (PaO_2_/FiO_2_ ≤ 200) that occurred within 48 h after intensive care unit (ICU) admission and was not related to other documented causes of acute respiratory dysfunction (22, 23). Postoperative AKI was defined as an increase from baseline of ≥0.3 mg/dl of postoperative creatinine or an increase of more than 1.5 times the preoperative creatinine within 48 h (24). Nosocomial infection was diagnosed as the criteria issued by Chinese Ministry of Health (25).

### Statistical analysis

The included patients were categorized into two groups based on whether to receive intraoperative RBC transfusion (IRT), namely, IRT (+) group and IRT (−) group. Continuous variables were expressed as mean and standard deviation or median and interquartile range depending on Gaussian distribution; categorical variables were reported as frequency and percentage. Likewise, group comparison of continuous variables was carried out using the Student’s *t*-test or Mann–Whitney U test, accordingly. Chi-squared test or Fisher’s exact test was used to test the difference in the distribution of categorical variables between groups. Contingency coefficient correlation test was used to explore the relationship of the binary categorical variables.

Multivariate logistic regression was applied to explore the predictors of intraoperative RBC transfusion. Age, female, preoperative atrial fibrillation, iron deficiency, hemoglobin, total bilirubin, hematocrit, and eGFR with a *P* value <0.1 in Table 1 were added into a stepwise variable selection, reporting odds ratio (OR) with 95% confidence interval (CI) for the independent predictors identified. Then a multivariable competing risk analysis model for postoperative hypoxemia was performed using factors chosen for clinical reasons or baseline variables with differences, including age, female, BMI, preoperative atrial fibrillation, iron deficiency, hemoglobin, total bilirubin, hematocrit, eGFR, CPBT, ACCT, surgical types, preoperative LVEF and intraoperative RBC transfusion. Moreover, the receiver operating characteristic (ROC) curve and the area under the curve (AUC) were estimated to assess the predictive validity of the logistic regression model.

**TABLE 1:** Baseline characteristics of the two groups.

Variable	Overall (*n* = 345)	IRT (+) (*n* = 84)	IRT (−) (*n* = 261)	*P*-value
Age (years)	59.7 ± 10.7	61.7 ± 10.1	59.0 ± 10.9	0.046
Female	158 (45.8)	62 (73.8)	96 (36.8)	< 0.001
BMI (kg/m^2^)	24.6 ± 3.9	25.2 ± 3.9	24.5 ± 3.8	0.127
**Comorbidities**				
Hypertension	108 (31.3)	31 (36.9)	77 (29.5)	0.203
Diabetes	24 (7.0)	7 (8.3)	17 (6.5)	0.569
Stroke	27 (7.8)	8 (9.5)	19 (7.3)	0.505
Preoperative atrial fibrillation	105 (30.4)	32 (38.1)	73 (28.0)	0.079
Smoking	24 (7.0)	3 (3.6)	21 (8.0)	0.248
Drinking	14 (4.1)	2 (2.4)	12 (4.6)	0.563
Iron deficiency	100 (29.0)	38 (45.2)	62 (23.8)	< 0.001
NYHA functional class ≥ III	315 (91.3)	77 (91.7)	238 (91.2)	0.892
**Surgical types**				0.100
Isolated mitral valve replacement	175 (50.7)	43 (51.2)	132 (50.6)	
Isolated aortic valve replacement	91 (26.4)	26 (31.0)	65 (24.9)	
Isolated tricuspid valve replacement	30 (8.7)	3 (3.6)	27 (10.3)	
Mitral and aortic valve replacement	48 (13.9)	11 (13.1)	37 (14.2)	
Mitral, aortic, and tricuspid valve replacement	1 (0.3)	1 (1.2)	0 (0.0)	
**Laboratory tests**				
PT(s)	13.9 ± 5.0	13.4 ± 3.4	14.0 ± 5.5	0.201
APTT(s)	29.6 ± 6.0	29.8 ± 6.5	29.6 ± 5.8	0.723
TT(s)	18.6 ± 3.9	18.6 ± 3.8	18.6 ± 3.9	0.876
Fibrinogen (g/L)	2.68 ± 0.81	2.74 ± 0.96	2.66 ± 0.75	0.473
Hemoglobin (g/L)	138.8 ± 11.4	132.9 ± 9.7	140.8 ± 11.2	< 0.001
Platelet count (×10^9^/L)	170.9 ± 54.0	171.5 ± 63.0	170.7 ± 50.9	0.918
ALT (U/L)	31.8 ± 25.4	32.7 ± 30.4	31.4 ± 23.7	0.724
Total bilirubin (μmol/L)	19.5 ± 15.6	17.0 ± 11.1	20.2 ± 16.8	0.095
Hematocrit (%)	41.8 ± 3.5	40.4 ± 3.2	42.2 ± 3.5	< 0.001
eGFR [ml/(min⋅1.73 m^2^)]	90.1 ± 29.3	83.2 ± 22.2	92.3 ± 30.9	0.004
Albumin (g/L)	36.0 ± 7.7	35.5 ± 7.9	36.2 ± 7.6	0.499
HbA1c (%)	6.4 ± 1.3	6.5 ± 1.3	6.4 ± 1.3	0.436
Preoperative LVEF (%)	58.3 ± 8.9	58.1 ± 8.9	58.3 ± 7.7	0.781

IRT, intraoperative red blood cell transfusion; BMI, body mass index; NYHA, New York Heart Association; PT, prothrombin time; APTT, activated partial thromboplastin time; TT, thrombin time; ALT, alanine transaminase; eGFR, estimated glomerular filtration rate; HbA1c, hemoglobin A1c; LVEF, left ventricular ejection fraction.

All statistical analyses were performed with the SPSS software (ver. 22.0 for Windows; SPSS Inc., Chicago, IL, USA). GraphPad Prism software (ver. 8.0 for Windows; Inc, USA) was used to create the graphics. A difference was considered significant for 2-sided *P* value < 0.05.

## Results

**FIGURE 1:**
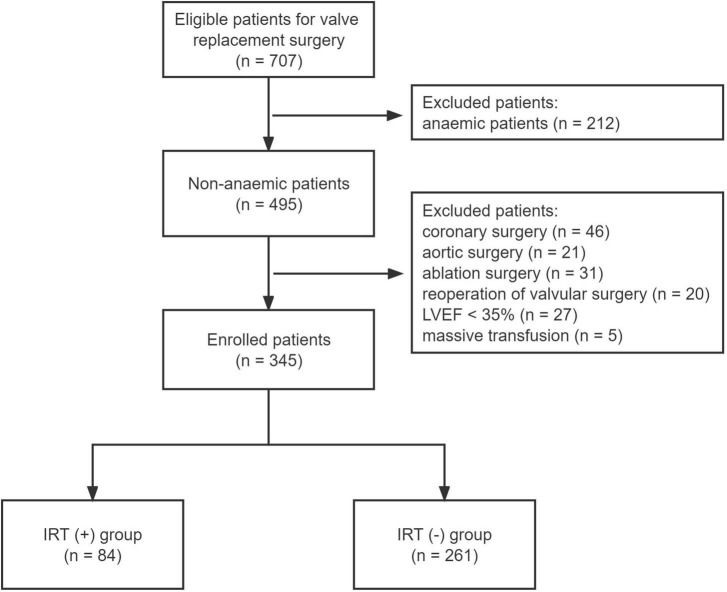
Flow chart of the study enrollment. LVEF, left ventricular ejection fraction; IRT, intraoperative red blood cell transfusion.

From January 2015 to December 2019, 707 consecutive patients underwent elective valvular replacements in our department. A total of 362 patients were excluded from the final analysis. The reasons for exclusion were shown in Figure 1. The enrolled 345 patients were finally divided into two groups: IRT (+) group (*n* = 84) and IRT (−) group (*n* = 261).

### Baseline characteristics and predictors of intraoperative red blood cell transfusion

Overall, there were 187 men (54.2%) and 158 women (45.8%) with a mean age of 59.7 ± 10.7 years. The mean preoperative hemoglobin was 138.8 ± 11.4 g/L. Most patients (91.3%) had New York Heart Association (NYHA) functional class III or IV. The baseline characteristics of patients were presented in Table 1. Compared with IRT (−) group, the patients of IRT (+) group were of older age (61.7 ± 10.1 vs. 59.0 ± 10.9 years, *P* = 0.046), lower level of hemoglobin (132.9 ± 9.7 vs. 140.8 ± 11.2 g/L, *P* < 0.001) and more likely to be iron-deficient (45.2% vs. 23.8%, *P* < 0.001).

Multiple logistic regression analysis for identification of the independent risk factors for intraoperative RBC transfusion was performed, variables including age, female, preoperative atrial fibrillation, iron deficiency, hemoglobin, total bilirubin, hematocrit, and eGFR with *P* value <0.1 at the univariate analysis in Table 2. Multivariate logistic regression model reported the independent predictors for intraoperative RBC transfusion were female (OR = 3.13, 95% CI: 1.73–5.93, *P* < 0.001), iron deficiency (OR = 1.97, 95% CI: 1.13–3.42, *P* = 0.017), and hemoglobin (OR = 0.96, 95% CI: 0.93–0.98, *P* < 0.001). The ROC curve of the multivariate logistic regression model illustrated the independent factors had an AUC of 0.760 (95% CI: 0.699–0.820) (Supplementary Figure 1), showing a significant predictive value for intraoperative RBC transfusion (*P* < 0.001). Hemoglobin exerted a “protective” effect for intraoperative RBC transfusion, which was negatively correlated with intraoperative RBC volume (*R* = −0.269, *P* < 0.001). Additionally, the serum ferritin level of IRT (+) group was significantly lower (168.0 ± 120.7 vs. 237.1 ± 148.0 ng/ml, *P* < 0.001) (Supplementary Figure 2).

**TABLE 2:** Multiple logistic regression for intraoperative RBC transfusion.

Variable	Univariate logistic regression		Multivariate logistic regression	
	OR (95% CI)	*P*-value	OR (95% CI)	*P*-value
Age (years)	1.02 (1.00–1.05)	0.047	–	
Female	4.84 (2.80–8.38)	< 0.001	3.13 (1.73–5.67)	< 0.001
Preoperative atrial fibrillation	1.59 (0.95–2.66)	0.081	–	
Iron deficiency	2.65 (1.58–4.44)	< 0.001	1.97 (1.13–3.43)	0.017
Hemoglobin (g/L)	0.93 (0.91–0.96)	< 0.001	0.96 (0.93–0.98)	0.001
Total bilirubin (μmol/L)	0.98 (0.96–1.00)	0.085	–	
Hematocrit (%)	0.85 (0.79–0.92)	< 0.001	–	
eGFR [ml/(min⋅1.73 m^2^)]	0.99 (0.98–1.00)	0.014	–	

eGFR, estimated glomerular filtration rate; OR, odds ratios; CI, confidence intervals.

### Relationship of intraoperative red blood celltransfusion and other blood products transfusion

Over the IRT (+) group, there were 50 cases (59.5%) getting transfusion of FFP, which was significantly higher than that of IRT (−) group (*P* < 0.001). Similarly, the incidences of platelet and cryoprecipitation transfusion in surgery were significantly higher in IRT (+) group (*P* < 0.001). Interestingly, the incidence of postoperative RBC transfusion seemed to be significantly increased after intraoperative RBC transfusion (*P* = 0.001) (Supplementary Table 1).

**FIGURE 2:**
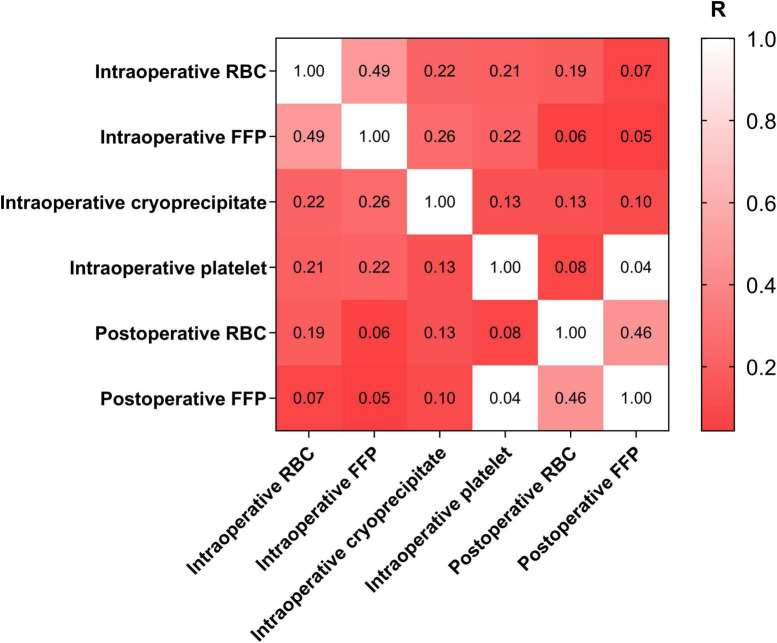
Correlations of different blood products. RBC, red blood cell; FFP, fresh frozen plasma.

The correlations of different blood products transfusion were shown in Figure 2. Notably, intraoperative RBC transfusion was moderately correlated with intraoperative FFP transfusion (Phi: 0.488, *P* < 0.001). Similarly, intraoperative RBC transfusion was significantly correlated with postoperative RBC transfusion (Phi: 0.185, *P* = 0.001).

### Impact of intraoperative red blood cell transfusion on in-hospital clinical outcomes

All the 345 patients successfully received the procedures. Overall, the median CPBT was 84.0 (73.0, 97.0) min, whereas the median ACCT was 57.0 (48.0, 70.0) min, which showed no difference between the two groups. The in-hospital mortality was 2.3% (8/345). When comparing all outcomes between the groups, it was found that IRT (+) group had a higher mortality (6.0% vs. 1.1%, *P* = 0.033) and an increased rate of postoperative hypoxemia (9.5% vs. 2.7%, *P* = 0.007). Importantly, postoperative hypoxemia was positively correlated with in-hospital mortality (Phi: 0.156, *P* = 0.004). MACCE, postoperative AKI, and nosocomial infection were not significantly different in the groups. The other outcomes were summarized in Table 3. In addition, patients in IRT (+) group were likely to get more drainage on the operation day compared with IRT (−) group (480 ± 230 vs. 361 ± 205 ml, *P* < 0.001), as well as on the first and second day after operation (*P* < 0.05) (Supplementary Figure 3).

**TABLE 3:** In-hospital clinical outcomes of the groups.

Variable	Overall (*n* = 345)	IRT (+) (*n* = 84)	IRT (−) (*n* = 261)	*P*-value
In-hospital mortality	8 (2.3)	5 (6.0)	3 (1.1)	0.033
MACCE	16 (4.6)	5 (6.0)	11 (4.2)	0.510
Duration of ventilator ≥ 48h	35 (10.1)	13 (15.5)	22 (8.4)	0.063
Postoperative hypoxemia	15 (4.3)	8 (9.5)	7 (2.7)	0.007
Postoperative AKI	16 (4.6)	5 (6.0)	11 (4.2)	0.510
Nosocomial infection	9 (2.6)	2 (2.4)	7 (2.7)	0.880
re-exploration for bleeding	3 (0.9)	0 (0.0)	3 (1.1)	0.756
Postoperative LVEF (%)	57.8 ± 8.6	57.3 ± 10.2	58.0 ± 8.1	0.580

IRT, intraoperative red blood cell transfusion; MACCE, major adverse cardiovascular and cerebrovascular events; AKI, acute kidney injury; LVEF, left ventricular ejection fraction.

**FIGURE 3:**
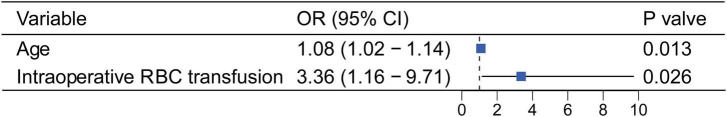
Forest plot depicting multivariate logistic regression results for the postoperative hypoxemia. OR, odds ratio; CI, confidence interval.

Given postoperative hypoxemia was likely to exacerbate in-hospital mortality, a multivariate logistic regression model was performed to determine the association of postoperative hypoxemia with intraoperative RBC transfusion. After adjusting for age, female, BMI, preoperative atrial fibrillation, iron deficiency, hemoglobin, total bilirubin, hematocrit, eGFR, CPBT, ACCT, surgical types, LVEF and intraoperative RBC transfusion, intraoperative RBC transfusion was confirmed as an independent risk factor for postoperative hypoxemia (OR = 3.36, 95% CI: 1.16–9.71, *P* = 0.026; Figure 3). The ROC curve of the multivariate logistic regression model also showed a significant prediction power for postoperative hypoxemia (*P* < 0.001), with an AUC of 0.764 (95% CI: 0.627–0.902) (Supplementary Figure 4).

## Discussion

Cardiac surgery is frequently accompanied by a large amount of blood loss, which leads to a great demand for allogeneic transfusion. Allogeneic RBC transfusion should be considered as one of the most effective methods to cure perioperative poor perfusion even when we focused on the population of non-anemic patients who underwent cardiac surgery. In this retrospective study, the proportion of intraoperative RBC transfusion reached 24.3% in non-anemic patients undergoing valve replacement. As was investigated, iron deficiency, female, and hemoglobin were found to be clinically relevant to intraoperative RBC transfusion in our study. Of note, hemoglobin was also shown as a protector of intraoperative RBC transfusion, which was consistent with previous studies (26). A higher concentration of hemoglobin could enhance the oxygen-carrying capacity of blood, which could obviously improve tolerance to blood loss. While preoperative iron deficiency and female have already been reported to be associated with RBC transfusion in elective cardiac surgery (27–29). We further validated that preoperative iron deficiency and female were also the independent predictors of intraoperative RBC transfusion in non-anemic patients undergoing valve replacement surgery. Indeed, iron deficiency was one of the main causes of preoperative anemia in patients undergoing cardiac surgery, which remained high prevalence in non-anemic group as well (30). The etiology of iron deficiency in cardiac surgery patients involves insufficient iron uptake, an increase in hepcidin synthesis, inhibition of erythropoietin synthesis, and decreased sensitivity of erythroblasts to erythropoietin (31), which leads to poor preoperative hemoglobin level. Several studies have shown that iron deficiency was closely related to the increase in perioperative blood transfusion in cardiac surgery, fatigue, and mortality (21, 28, 29). But most of the participants in these studies were anemic. However, Miles et al. (32) reported that there was weak evidence of an association between non-anemic iron deficiency and the outcomes of cardiac surgery, including mortality, MACCE, and hospital length of stay, after controlling for confounding variables, such as preoperative age, sex, renal function, EuroSCORE, and hemoglobin. We tentatively propose that iron deficiency might still result in an insufficient blood reserve in non-anemic patients, making it difficult to maintain the fluctuation of blood volume. Therefore, the patients with iron deficiency had a greater chance of suffering intraoperative RBC transfusion.

Notably, intraoperative RBC transfusion was used as a treatment of tissue hypoxia. However, it seemed to indicate that the patients suffered higher rate of postoperative hypoxemia after intraoperative RBC transfusion, which was correlated with in-hospital mortality. After adjustment, intraoperative RBC transfusion was identified as an independent risk factor of postoperative hypoxemia. Indeed, postoperative hypoxemia is one of the most common complications after cardiac surgery, and is associated with a higher risk of morbidity and mortality (33, 34). In similarity, previous studies reported that intraoperative RBC transfusion was associated with high occurrence of postoperative hypoxemia in surgery for acute aortic dissection (33, 35), supporting our findings. Furthermore, Wang et al. (36) also verified intraoperative RBC transfusion as one of the independent risk factors of severe hypoxemia in all kinds of cardiac surgeries, among which isolated valve surgery accounted for 54%. Recently, a consequence of transfusion-related immune modulation may partially explain the adverse effects of blood transfusions (37, 38). Importantly, intraoperative RBC transfusion was indeed correlated with transfusion of other blood products, which implied that poor prognosis might be the consequence of multiple blood products transfusion. In addition, it was also revealed that intraoperative transfusion of blood products such as RBC, FFP and platelets could also lead to a significant increase of postoperative infection, acute lung injury and acute kidney injury in cardiac surgery (5, 39, 40). Thus, inappropriate transfusion would not benefit the patients undergoing cardiac surgery (2, 41).

It is critical to improve perioperative blood management to reduce post-complications. Actually, in many institutions, the implementation of cell savers had relatively completed blood management to reduce allogeneic transfusion and alleviated blood shortage accordingly (42). In the meantime, with the emergence of the restrictive transfusion, the criteria for triggering blood transfusions remains controversial (43). Zeroual et al. (44) suggested a restrictive transfusion strategy adjusted with central venous oxygen saturation after cardiac surgery, which might be also applied intraoperatively. Additionally, improvement in preoperative status can also reduce the intraoperative transfusion of blood products. Recently, a randomized prospective trial indicated that intravenous iron with vitamin B12 and folic acid on the day before the operation would significantly reduce allogeneic transfusion in non-anemic patients with iron deficiency (45). Several retrospective studies demonstrated that intravenous iron could significantly increase hemoglobin level, reduce fatigue, and improve physical endurance in non-anemic patients undergoing cardiac surgery (46, 47). These studies also supported our findings on the influence of iron deficiency on non-anemic patients. However, the role of iron metabolism in acute hemorrhage and hemodilution is still debated widely, which needs further exploration.

There are several limitations in our study as followed. Firstly, the main limitation was its retrospective single-center design, implying that the external validity of our results might be limited. Secondly the sample size was not big enough to enhance our statistical validity to detect other significant differences in clinical outcomes. Thirdly, as mentioned above, the impact of intraoperative RBC transfusion on in-hospital clinical outcomes might be interfered with transfusion of other blood products. Subgroup analysis based on different blood products should be needed when more patients are included. Finally, the study was too homogeneous in terms of the diseases, and the findings needed further validation in other disease conditions, such as congenital heart disease and coronary artery disease.

## Conclusion

Intraoperative RBC transfusion could significantly increase the risk of postoperative hypoxemia in non-anemic adults undergoing isolated valve replacement, which might lead to an increase of in-hospital mortality consequently. Iron deficiency and female were indicated as the independent predictors affecting intraoperative RBC transfusion. Thus, treatment of preoperative iron deficiency could be an effective method to reduce usage of blood products in subsequent clinical work. Thereby, it is valuable to follow up long-term outcomes of intraoperative transfusion in future for the establishment of evidence-based blood management.

## Data availability statement

The datasets generated and analyzed during the current study are not publicly available at this time as the data also forms part of another ongoing study but are available from the corresponding author on reasonable request. Requests to access the datasets should be directed to RZ, 862722617@qq.com.

## Ethics statement

The studies involving human participants were reviewed and approved by the Ethical Committees of Shanghai General Hospital. Written informed consent for participation was not required for this study in accordance with the national legislation and the institutional requirements.

## Author contributions

MY, DY, and RZ designed the research. RZ and DQ collected the data. RZ, HL, SS, and FS performed data analyses. RZ, DQ, and HL wrote the manuscript. LC and MY revised the manuscript. ZW, SS, and FS helped optimize the research and proofread the manuscript. All authors contributed to manuscript revision, read, and approved the submitted version.
