# Supplementary material for: Clinical presentation and in-hospital outcomes of intraoperative red blood cell transfusion in non-anemic patients undergoing elective valve replacement
Source: Front Cardiovasc Med. 2022 Nov 22;9:1053209. doi: 10.3389/fcvm.2022.1053209 (PMC9723323; doi:10.3389/fcvm.2022.1053209)
Supplement: Supplementary file 1 [file Data_Sheet_1.docx]

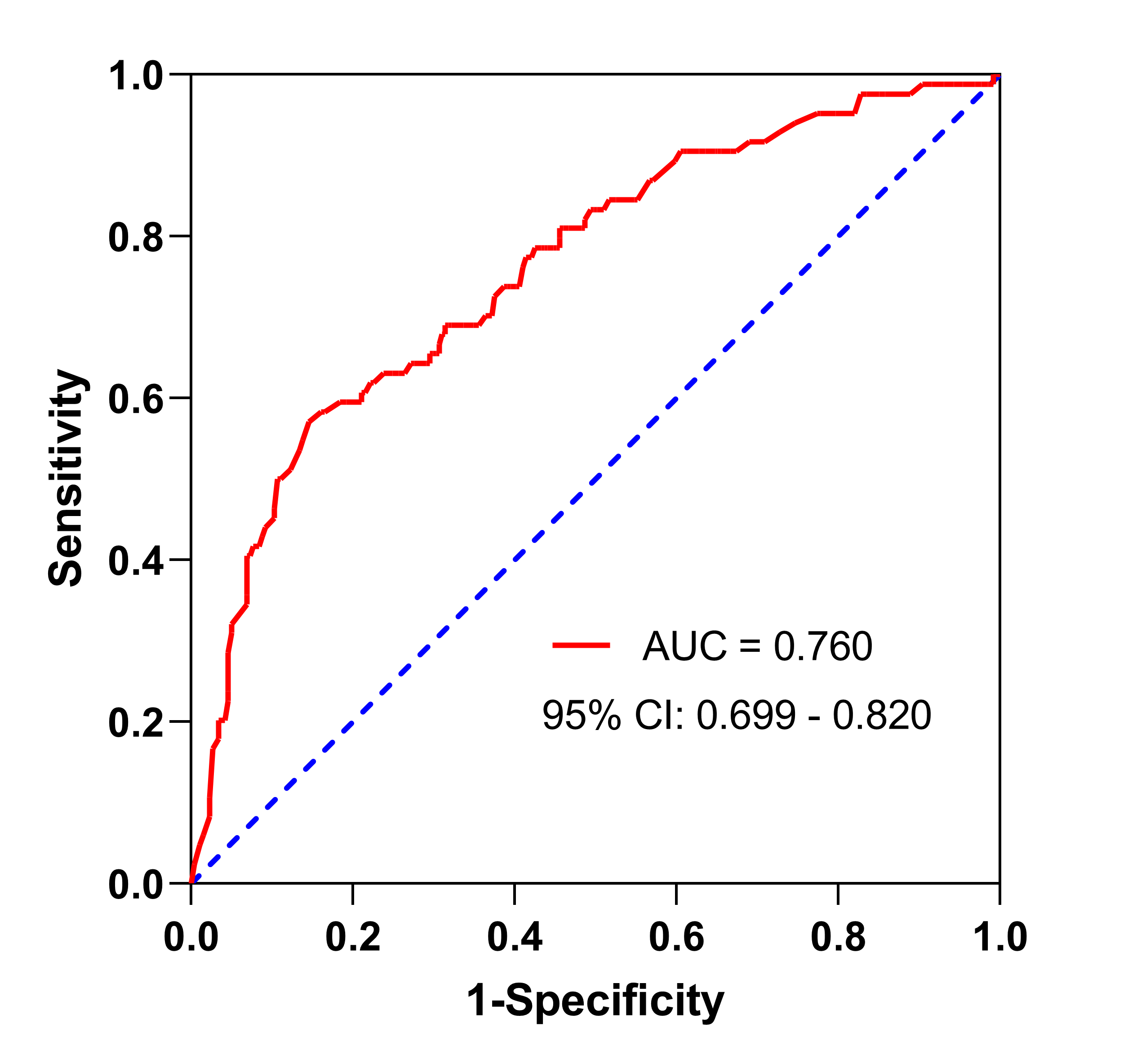


Supplementary Figure 1. Receiver operating characteristic curves for the logistic regression model of intraoperative RBC transfusion. AUC, area under the receiver operating characteristic curve; CI, confidence interval.


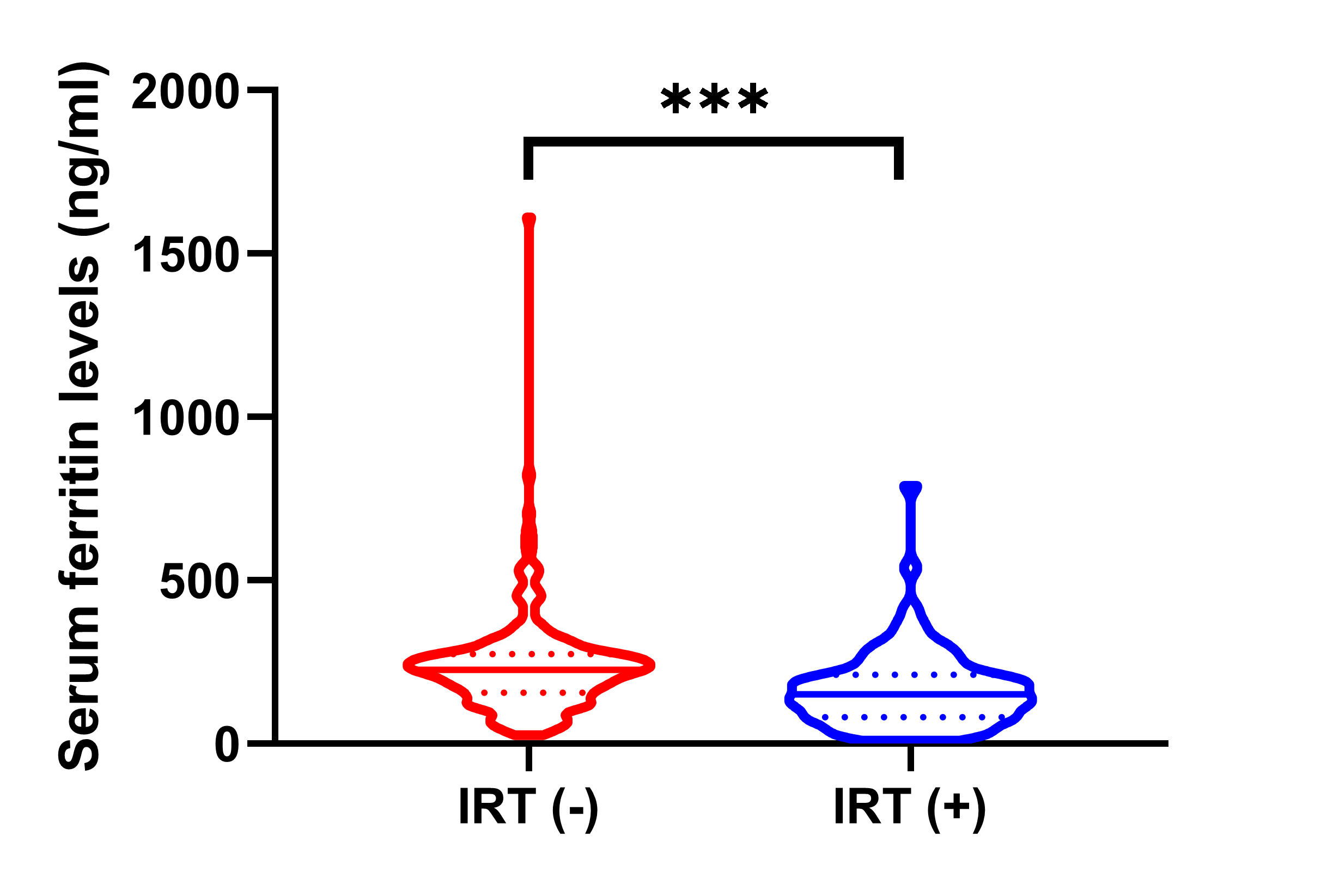


Supplementary Figure 2. Distribution of serum ferritin levels in two groups. IRT: intraoperative red blood cell transfusion. ^***^ *P* < 0.001.


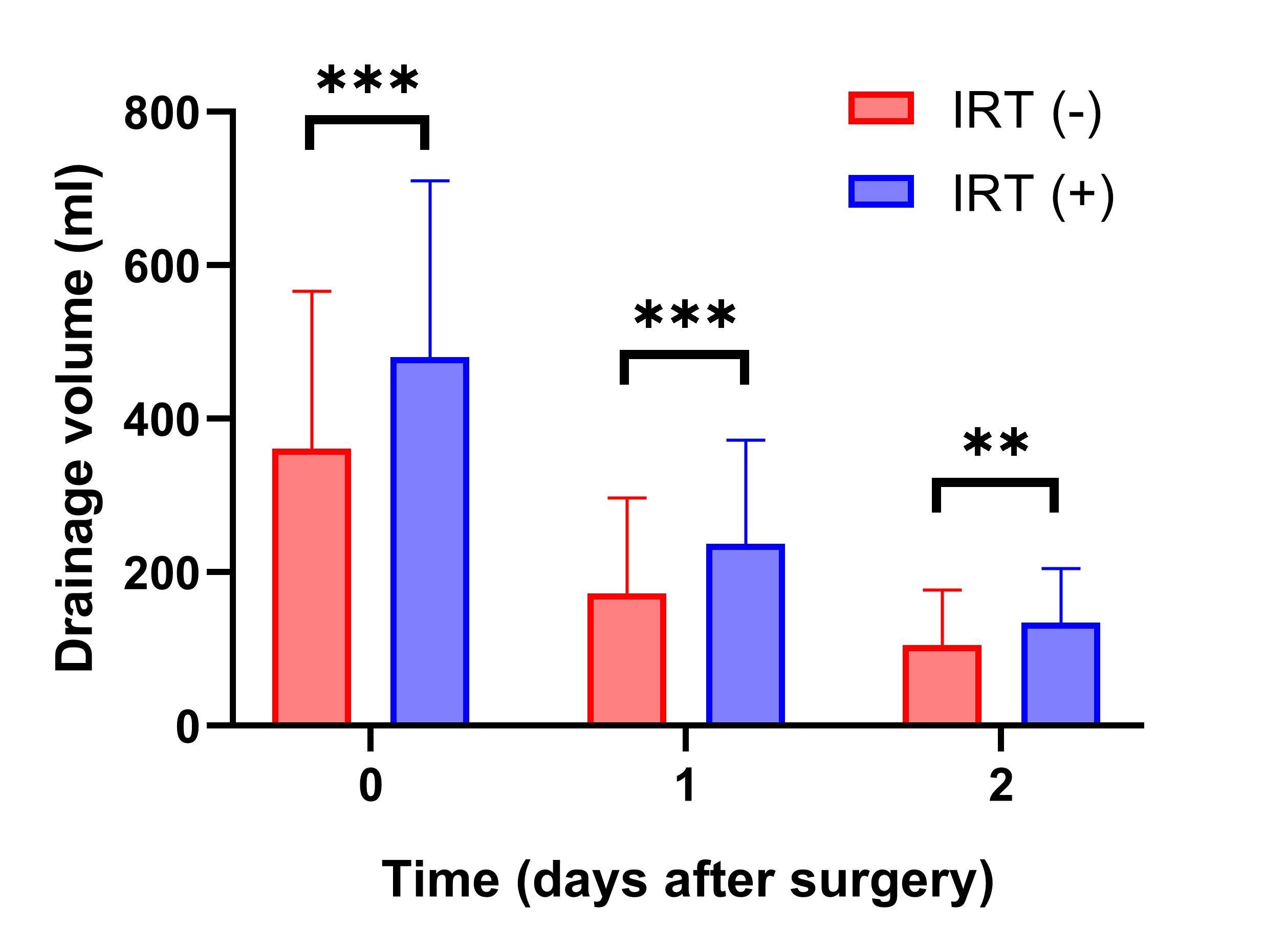


Supplementary Figure 3. Drainage within 2 days after surgery. IRT: intraoperative red blood cell transfusion. ^***^ *P* < 0.001; ^**^ *P* < 0.01.


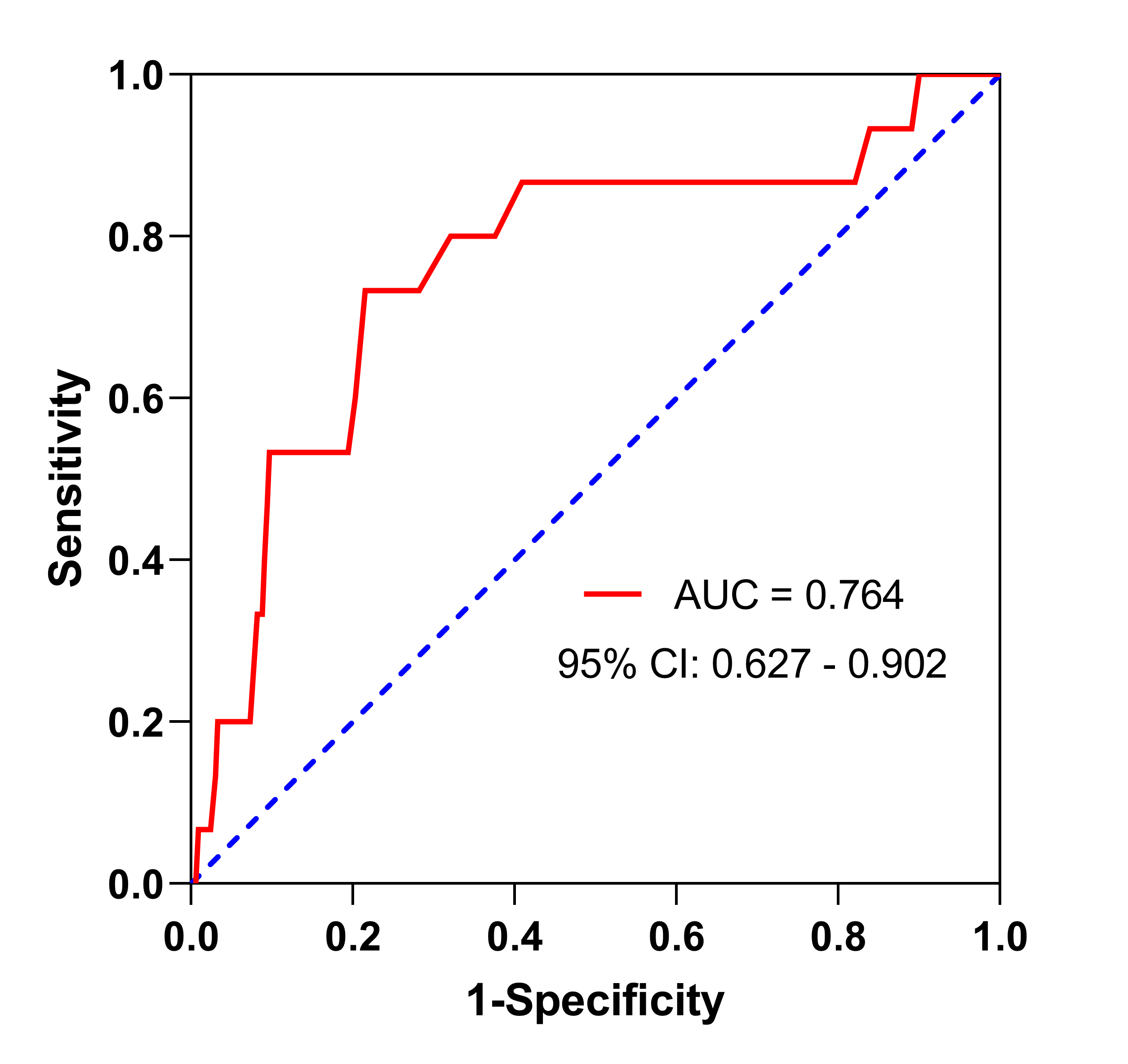


Supplementary Figure 4. Receiver operating characteristic curves for the logistic regression model of postoperative hypoxemia. AUC, area under the receiver operating characteristic curve; CI, confidence interval.
