# Supplementary material for: Clinical presentation and in-hospital outcomes of intraoperative red blood cell transfusion in non-anemic patients undergoing elective valve replacement
Source: Front Cardiovasc Med. 2022 Nov 22;9:1053209. doi: 10.3389/fcvm.2022.1053209 (PMC9723323; doi:10.3389/fcvm.2022.1053209)
Supplement: Supplementary file 2 [file Table_1.DOCX]

Supplementary table 1. Different blood products transfusion in the groups.

| Variable | Overall  （n = 345） | IRT (+)  (n = 84) | IRT (-)  (n = 261) | *P* value |
| --- | --- | --- | --- | --- |
| Intraoperative FFP transfusion | 80 (23.2) | 50 (59.5) | 30 (11.5) | ＜0.001 |
| Intraoperative platelet transfusion | 25 (7.2) | 14 (16.7) | 11 (4.2) | ＜0.001 |
| Intraoperative cryoprecipitation transfusion | 26 (7.5) | 15 (17.9) | 11 (4.2) | ＜0.001 |
| Postoperative RBC transfusion | 60 (17.4) | 25 (29.8) | 35 (13.4) | 0.001 |
| Postoperative FFP transfusion | 50 (14.5) | 16 (19.0) | 34 (13.0) | 0.173 |

IRT: intraoperative red blood cell transfusion; FFP: fresh frozen plasma; RBC: red blood cell.
